# Primitive-Reflex Profiling After Sport-Related Concussion: An Athlete-Centered Framework for Testing a Candidate Behavioral State Marker

**DOI:** 10.3390/brainsci16090968

**Published:** 2026-09-14

**Authors:** Erzsébet Stephens-Sarlós, Patrick Stephens, Attila Szabo

**Affiliations:** Faculty of Health and Sport Sciences, Széchenyi István University, 9026 Győr, Hungary

**Keywords:** primitive reflexes, sport-related concussion, sensorimotor gating, fronto-subcortical control, vestibular–reticulospinal function, functional near-infrared spectroscopy, within-athlete design, staged validation

## Abstract

**Highlights:**

**What are the main findings?**

- Standardized primitive-reflex profiling may offer a low-cost candidate behavioral state marker of sensorimotor change after sport-related concussion, particularly when evaluated against each athlete’s own pre-injury baseline.
- The proposed validation framework combines serial reflex assessment with matched uninjured controls and multimodal measures. Lateralized grasp-related reflexes and fNIRS provide a specific, falsifiable test case.

**What are the implications of the main findings?**

- Primitive-reflex profiling should currently be used only as an experimental research outcome alongside guideline-concordant concussion assessment—not as a diagnostic biomarker, treatment-selection tool, or return-to-sport criterion.
- Clinical development would require reliable change beyond measurement error, temporal coherence with recovery, added prognostic value beyond established assessments, and independent replication.

**Abstract:**

Direct prospective evidence that primitive-reflex expression changes after sport-related concussion (SRC) is currently very limited. This Perspective therefore proposes a deliberately restricted context of use: standardized primitive-reflex profiling as an experimental, low-cost candidate behavioral state marker to be tested within athletes across recovery, not as an established diagnostic biomarker or a return-to-sport criterion. The standardized 18-reflex protocol underlying this framework demonstrated excellent inter-rater (κ = 0.912–1.000) and intra-rater (κ = 0.912–0.983) reproducibility in the reported 565-participant validation sample; however, athlete-specific reliability, responsiveness to SRC, construct validity, prognostic utility, and treatment sensitivity remain unestablished. We distinguish three testable candidate mechanisms—fronto-subcortical stopping-network dysfunction, altered sensorimotor/afferent gating, and vestibular–reticulospinal dysregulation—and specify findings that would support or weaken each account. A staged research program begins with sport-specific reliability and feasibility, proceeds to prospective within-athlete change beyond measurement error, and only then evaluates mechanistic convergence, incremental prognostic value, and intervention sensitivity. The full 18-reflex profile (30 bilateral or non-lateralized measurements) should remain the primary level of analysis. A summed primitive-reflex burden is retained only as a descriptive summary and must not substitute for reflex-specific analysis. Palmar grasp and Babkin-related responses provide a focused, falsifiable test case for relating lateralized reflex expression to premotor and supplementary motor hemodynamics during force-matched grasping. Progression between stages requires prespecified success criteria, independent replication, and explicit stopping rules. Until those conditions are met, primitive-reflex profiling should remain an experimental outcome embedded within guideline-concordant concussion care.

## 1. Introduction

Sport-related concussion (SRC) is a mild traumatic brain injury produced by direct or transmitted biomechanical forces. Contemporary consensus recommends a multimodal clinical assessment that integrates symptoms, cognition, balance, vestibular–oculomotor function, neurological examination, exercise tolerance, and staged return to activity [1,2]. Targeted rehabilitation is supported for selected persisting symptoms, but current evidence does not establish primitive-reflex testing or reflex-directed treatment as standard concussion care [1,3].

Recovery is not necessarily synchronous across domains: symptoms may improve while physiological abnormalities persist [4]. Studies using transcranial magnetic stimulation (TMS) report alterations in motor-system excitability and inhibitory function after concussion [5,6], and autonomic dysfunction and exercise intolerance are also documented [7,8]. The resulting heterogeneity is one reason that sport-concussion research emphasizes common data elements and repeated, multidomain assessment rather than reliance on a single test [9].

Direct prospective evidence that primitive-reflex expression changes following SRC is currently very limited. Evidence from other forms of brain injury and from studies demonstrating altered sensorimotor regulation after concussion provides biological plausibility, but does not establish an SRC-related change in primitive reflexes. Accordingly, the proposed relationship should be regarded as a testable hypothesis rather than an established finding.

Throughout this Perspective, “primitive-reflex profiling” is an operational umbrella term for a heterogeneous battery of 18 primitive, transitional, and postural responses. These responses differ in developmental characteristics, eliciting conditions, and neural organization. Their expression or suppression should not be assumed to share a single neurobiological mechanism. Cortical modulation may contribute, but its relative role and that of subcortical, brain-stem, and spinal processes require reflex-specific investigation. The demonstrated reproducibility of the examination protocol does not establish a common mechanism. Mechanistic evidence for each response would be needed before it could guide the selection of movement-based interventions.

This Perspective defines a narrow and falsifiable context of use: primitive-reflex profiling is proposed as an experimental candidate behavioral state marker for repeated within-athlete study. It is not proposed as a stand-alone diagnostic biomarker, a surrogate for tissue injury, a measure of γ-aminobutyric acid (GABA), or a return-to-sport decision rule. The proposal succeeds only if reflex-specific measures are reproducible in athletes, change beyond measurement error after SRC, recover coherently, converge with independently measured systems, add information beyond established assessments, and replicate independently.

## 2. Scope and Novelty of the Perspective

A previous lifespan Perspective developed the general hierarchical-gating model of primitive reflexes, proposed multimodal physiological correlation, and outlined bottom-up and top-down intervention axes [10]. Those general concepts are therefore not claimed as new in the present article. Here, we reformulate the model as a sport-specific, time-locked injury hypothesis that can be tested prospectively with repeated preseason baselines, a defined SRC event, serial within-athlete measurements, matched uninjured controls, and explicit failure criteria. These design features permit stronger tests of temporal association and of prespecified mechanistic predictions. They do not by themselves establish causality.

The distinct contribution is deliberately narrower: a standardized 18-reflex, 30-measure athlete profile; repeated preseason baselines; injury-linked within-athlete change that must exceed measurement error; preservation of reflex- and side-specific data; competing, falsifiable neural predictions; and a stage-gated program that requires psychometric qualification before mechanistic, prognostic, or intervention claims. Neither the Amsterdam consensus nor a contemporary systematic review of targeted concussion interventions includes primitive-reflex profiling as a validated assessment or a reflex-specific treatment algorithm [1,3].

The NERD model already provides a systems-level account of reflex-circuit dysfunction in persistent post-concussion symptoms and outlines longitudinal, neuroimaging, prediction, intervention, and falsification studies [11]. The present Perspective does not claim those broad directions as novel. Its added value is the sport-specific operationalization described above, including the requirement for within-athlete change beyond error and reflex-specific progression criteria before higher-level claims are attempted. The limited clinical literature must also be interpreted cautiously. Ziaks et al. reported findings from 82 patients with protracted concussion who received an integrative rehabilitation protocol combining reflex-related, visual, vestibular, and other components [12]. The retrospective, uncontrolled, and multimodal design could not isolate an effect attributable specifically to the primitive-reflex component. A later review likewise concluded that the scarcity of controlled studies prevents firm conclusions about mechanism or efficacy [13].

## 3. Systems-Neuroscience Rationale

### 3.1. Distributed Hierarchical Control

Primitive reflexes are early-developing, stimulus-linked sensorimotor responses. Their reappearance in neurological disease has often been described as a frontal-release phenomenon [14,15,16], but that label should not be mistaken for a unitary lesion mechanism. The observed response integrates afferent input, spinal and brain-stem circuitry, subcortical selection, cortical preparation and inhibition, biomechanical state, attention, and examiner technique within dynamically regulated networks [17]. Developmental work also indicates that grasping reflects maturation of distributed sensorimotor connectivity rather than a single descending switch [18].

We therefore use the operational phrase “context-dependent descending sensorimotor gating dysregulation.” This denotes a failure to select, scale, or suppress an elicited motor pattern under defined conditions. It does not identify which anatomical level is causal. This wording preserves a systems hypothesis while allowing frontal, subcortical, vestibular–reticulospinal, spinal, peripheral, and non-neural explanations to be compared.

### 3.2. Evidence Must Be Separated from Mechanism

Two observations motivate—but do not prove—the proposal. First, primitive or frontal-release signs occur in several neurological conditions and may relate to cognition or function [14,15,16]. Second, concussion studies report motor-cortical and network-level physiological abnormalities [5,6]. A causal link between those studies has not been demonstrated. Primitive-reflex scores are therefore not direct measures of cortical inhibition, GABA concentration, structural injury, or any single pathway.

This distinction is especially important because excitation–inhibition balance is dynamically regulated across networks [17], and magnetic resonance spectroscopy, TMS, electroencephalography (EEG), functional near-infrared spectroscopy (fNIRS), and behavior index different biological quantities. Age-related trajectories of cortical GABA measured with spectroscopy [19], for example, cannot be used to infer GABA from a bedside reflex score. Convergence across modalities would strengthen a systems account. Disagreement would constrain or falsify it.

### 3.3. Candidate Mechanisms and Discriminating Predictions

Three non-exclusive neural mechanisms are prioritized. Each predicts a different pattern of convergent findings. A fourth, non-specific measurement account must always be retained. Mechanism is not assigned from the total reflex score. It should be tested by considering the reflex involved, the eliciting condition, laterality, time course, and an independently measured physiological system.

#### 3.3.1. Fronto-Subcortical Stopping-Network Dysfunction

The palmar grasp response is biologically plausible as a fronto-subcortical test case. In 491 patients with hemispheric lesions, De Renzi and Barbieri found grasp responses especially often after medial frontal and deep lesions [20]. In idiopathic normal-pressure hydrocephalus, grasp severity was associated with right fronto-subcortical white-matter abnormalities [21]. These findings support circuit plausibility outside SRC. They do not demonstrate that concussion causes a grasp reflex. Frontal-release signs after more severe traumatic brain injury have also been related to subacute cognitive and functional impairment, but those data cannot be generalized directly to mild SRC [22].

If fronto-subcortical stopping-network dysfunction contributes after SRC, the expected pattern is a new, increased, or lateralized grasp response relative to the athlete’s own stable baseline, accompanied by an independently abnormal stopping or motor-control measure. The primary fNIRS prediction is a reduced task-evoked contralateral premotor/supplementary motor area (SMA) oxygenated-hemoglobin response during force-matched voluntary grasping. Hemispheric lateralization is a secondary measure and must be interpreted from the separate hemispheric responses, as detailed in Section 4. Independent candidates include altered TMS indices such as the cortical silent period or long-interval intracortical inhibition and impaired fronto-subcortical structural or functional connectivity. Increased hemodynamic recruitment is an alternative finding, not confirmation of the primary attenuation hypothesis. The account is weakened if reliable reflex change repeatedly occurs without any independently measured fronto-subcortical deviation or recovery coupling.

#### 3.3.2. Altered Sensorimotor or Afferent Gating

Concussion-related TMS findings do not justify a simple “loss of inhibition” narrative. Longer cortical silent periods and increased long-interval intracortical inhibition have been described in athletes with a history of concussion, including those who were asymptomatic at assessment [23,24], whereas afferent-inhibition measures may remain normal [24]. A sensorimotor-gating mechanism would instead predict reflex-specific changes in stimulus threshold, response probability or slope, stimulus-locked surface electromyography, task-dependent cutaneous or H-reflex modulation, or short- and long-latency afferent inhibition. Stable, normal afferent-gating and reflex-gain measures despite reproducible behavioral reflex change would weaken this account.

#### 3.3.3. Vestibular–Reticulospinal Dysregulation

This account is most relevant to head-position–dependent tonic responses, Moro-like responses, or asymmetric tonic neck patterns. The immediate “fencing response” after moderate experimental brain injury has been interpreted as an asymmetric tonic-neck-like response involving the lateral vestibular nucleus [25]. It is an acute sign in a moderate-injury model, not evidence for persistent tonic reflexes after mild SRC. Support after SRC would require coupling with vestibular–ocular reflex measures, vestibular–ocular motor screening (VOMS), head-turn–evoked limb electromyography, sensory-integration balance testing, or startle/postural measures. Normal and stable vestibular–reticulospinal measures, particularly when only cortical measures change, would weaken this mechanism.

#### 3.3.4. Non-Specific or Measurement-Related Change

Pain, anxiety, arousal, fatigue, medication, musculoskeletal limitation, expectancy, inconsistent stimulus delivery, and unblinded scoring can all alter an observed response. This alternative is favored by poor test–retest stability, similar changes in uninjured controls, dependence on examiner knowledge, inconsistent video adjudication, lack of reflex specificity, or trajectories unrelated to recovery. Blinding, repeated baselines, standardized stimuli, raw side-specific recording, and prespecified covariates are therefore part of the biological test rather than optional methodological refinements. The mechanism-specific predictions and findings that would weaken each account are summarized in Table 1.

## 4. Grasp-Related Reflexes as a Focused Test Case

Palmar grasp and Babkin-related responses are selected as an initial test case because the stimulus can be standardized, the output is observable, laterality can be retained, and a force-matched voluntary grasp task can probe a related motor network. They are not assumed to represent the physiology of the other 16 reflexes. The adult lesion evidence summarized above [20,21] supports a stopping-network hypothesis specifically for grasp-related responses. It does not validate a global primitive-reflex burden construct.

fNIRS studies demonstrate that task-related cortical hemodynamics can be measured after concussion [26,27], but fNIRS does not measure neural inhibition directly. For the initial grasp-related test case, the primary directional hypothesis is that athletes with a new or increased unilateral grasp-related response after SRC will show an attenuated task-evoked oxygenated-hemoglobin (HbO) response in prespecified contralateral premotor and optically accessible SMA regions during force-matched voluntary grasping relative to their own preseason baseline and the change observed in matched uninjured controls. Support requires an effect in this direction that meets prospectively defined effect-size, precision, and multiplicity-adjusted statistical criteria. Coupling between reflex change and HbO attenuation, followed by a coherent return toward baseline, would provide additional support. Region selection, task contrast, and the primary outcome must be fixed before examining the data.

Hemispheric lateralization is secondary and must be interpreted alongside separate contralateral and ipsilateral estimates. Reduced lateralization caused by additional ipsilateral recruitment does not confirm the primary attenuation hypothesis. Increased contralateral or bilateral HbO is an alternative finding that may be compatible with compensation if it accompanies preserved task performance or recovery-related improvement. Increased HbO alone is insufficient to establish compensation. Altered effort, inefficient recruitment, systemic physiology, and neurovascular coupling remain competing explanations. A sufficiently precise null result excluding the prespecified meaningful attenuation, or a reproducible effect in the opposite direction, would weaken the primary directional hypothesis. Imprecise estimates remain inconclusive. A non-significant *p* value alone does not falsify the hypothesis.

For each reflex progressing to stage 3, standardized elicitation and scoring must be distinguished from voluntary reproduction of a related movement. Where anatomically meaningful and clinically safe, a secondary experiment may compare a voluntary reflex-congruent movement with a defined counter-pattern movement during fNIRS recording. Each comparison requires a reflex-specific hypothesis, counterbalanced task order, suitable rest or control conditions, and measurement or matching of force, rate, range of motion, task difficulty, and performance. The hemodynamic contrast would describe differential task-related recruitment. It would not show that reproducing the movement elicits the reflex or that the counter-pattern directly inhibits it.

The grasp task should control force, rate, hand order, fatigue, pain, handedness, prior concussion, and motor impairment. The optode montage should provide bilateral coverage of the prespecified, optically accessible cortical regions. Broader coverage may support exploratory analyses, but cannot sample the entire cortex or deep structures. Optode localization and cortical sensitivity should be documented. Optical density, signal-to-noise thresholds, short-separation regression, systemic physiology, motion correction, channel-level quality control, and correction for multiple testing should follow established fNIRS reporting practices [28]. Analyses should distinguish contralateral premotor/SMA regions of interest from exploratory channels and should report both HbO and deoxygenated-hemoglobin (HbR) estimates. These represent changes in hemoglobin concentrations, not separate measurements of arterial and venous oxygenation [28].

Reduced HbO cannot by itself localize the cortical source of reflex inhibition. Subsequent functional magnetic resonance imaging (fMRI) could assess anatomical and network convergence, but its blood-oxygen-level–dependent (BOLD) signal is also an indirect hemodynamic measure [29]. Agreement between fNIRS and fMRI would support convergent recruitment patterns, not establish an inhibitory mechanism. That inference requires independent, mechanism-sensitive neurophysiological evidence.

## 5. Measurement Model and Validation Requirements

### 5.1. Reflex Battery and Operational Administration

The standardized examination protocol contains 18 named reflex examinations: 12 are recorded bilaterally and 6 are recorded once rather than separately by side, yielding 30 measurements [30,31]. Table 2 provides an in-article operational overview of body position, eliciting maneuver, and recording laterality. The complete, versioned protocol is openly available in Mendeley Data and contains the step-by-step administration procedures, operational response criteria, contraindications and safety adaptations, demonstration materials, and examiner guidance required for standardized implementation [31]. Testing must occur in a quiet, safe environment, with standardized positioning and stimulus delivery, minimal cueing, and documentation of any safety or technical constraint.

Thus, Table 2 is not offered as a substitute for a measurement manual: it makes the examined domains and stimuli visible within the manuscript, whereas the cited Mendeley record supplies the exact positive motor-response criteria and standardized examiner procedures needed for replication [31]. The empirical reliability study then evaluates whether those prespecified procedures can be reproduced across and within raters [30].

### 5.2. Scoring, Side Specificity, and the Role of Total Burden

For each examinable response, the source protocol uses an ordinal score of 0 (not elicitable), 1 (partially elicitable), or 2 (fully elicitable). A code of 3 indicates that the item could not be examined because of a safety or technical constraint and must be treated as missing, not as a higher-severity score. When an observable response is ambiguous, the protocol permits one repeat and records the maximum response [30,31]. Athlete studies should also retain the raw behavioral description, side, trial, examiner, reason for missingness, and—where consent and governance permit—video for blinded adjudication.

The protocol has been empirically validated in a reported sample of 565 participants [30]. In the inter-rater subset (n = 259), Cohen’s κ ranged from 0.912 to 1.000. In the intra-rater subset (n = 306), κ ranged from 0.912 to 0.983. These values indicate excellent inter- and intra-rater reproducibility under the reported validation conditions and support the standardization of administration and scoring. They do not, however, establish sport-specific test–retest reliability, responsiveness to SRC, construct validity in athletes, prognostic utility, treatment sensitivity, or clinical decision validity. Each property must be tested separately in the intended population and context of use.

A total primitive-reflex burden (PRB) score can summarize the number and degree of elicitable responses and may reduce the number of global comparisons. Its use is justified only as a descriptive index of overall profile change. The 30 component measurements are ordinal, may differ in reliability and neural substrate, and cannot be assumed to form an interval scale or a single latent physiology. Therefore, individual reflexes and side-specific scores must always be preserved and reported. The PRB total alone must not determine mechanism, prognosis, treatment selection, or return-to-sport status. Primary reflexes and directions should be prespecified, and secondary reflex-wise analyses should control multiplicity. A full-battery PRB total should be calculated only when all 30 components are available. Unassessed items must not be assigned a severity score or treated as zero. Any prespecified reduced profile must identify its components and denominator, with longitudinal comparisons using the same assessed items.

### 5.3. Reliability, Measurement Error, and Responsiveness

Although the protocol has demonstrated excellent inter- and intra-rater reproducibility in the reported general validation sample [30], a candidate state marker must first be distinguished from stable trait variation within the target athletic population. Repeated preseason measurements should quantify day-to-day and examiner-related variance, practice or habituation, menstrual or hormonal context where relevant, sleep, fatigue, pain, medication, and training load. Agreement should be reported with reflex-appropriate statistics, confidence intervals, standard error of measurement (SEM), and smallest detectable change (SDC), not correlation alone.

Post-injury responsiveness is a different property from reliability. Individual change should exceed the athlete-specific or sample-derived SDC and should be contrasted with time-matched uninjured controls to separate injury effects from repeated-testing drift. Reliable-change methods have improved interpretation of post-concussion cognitive testing [32] and offer a transparent model for reflex outcomes, although the required error parameters must be established specifically for this battery.

## 6. A Sequential Athlete-Centered Research Program

The framework is intentionally sequential. Each stage addresses one inferential question and has a prespecified stopping rule. Failure at an earlier stage prevents escalation to more costly mechanistic tests, prognostic claims, or intervention studies.

### 6.1. Stage 1—Sport-Specific Reliability and Feasibility

Healthy athletes undergo at least two preseason examinations separated by a prespecified interval. Examiners are trained and blinded to prior scores. Video rescoring is used where feasible. Completion, adverse events, missingness, floor effects, inter- and intra-rater agreement, SEM, and SDC are estimated for every reflex and side. Reflex- and side-specific reliability and feasibility criteria must be defined in the study protocol and analysis plan before examining the study results. Prespecification should cover agreement and required precision, acceptable completion and missingness rates, assessment burden, and safety-related progression or stopping thresholds. Criteria must not be selected or relaxed in response to observed results. The program stops or the protocol is revised if these criteria are not met. A revised protocol requires renewed evaluation against prospectively defined criteria.

### 6.2. Stage 2—Prospective Within-Athlete Change After SRC

Clinical safety takes priority over research completeness, particularly in the acute phase after SRC. Research examinations must not delay or compromise guideline-concordant assessment and care [1,2]. Individual maneuvers should be performed only when the treating clinician considers them safe and clinically appropriate. An examination that cannot be performed safely must be deferred or discontinued and recorded as missing, with the reason and time point documented, rather than scored as abnormal or absent. Safety-related non-performance must not be interpreted as evidence of post-injury reflex change. Athletes with SRC are assessed as soon as clinically appropriate and then at prespecified recovery points, with the athlete’s own baseline as the primary comparator and matched uninjured athletes controlling for time and repeated testing. Change in safely obtained, comparable measurements must exceed SDC and show an injury-linked trajectory. Direct prospective evidence begins at this stage. Without it, mechanistic or clinical claims do not advance.

### 6.3. Stage 3—Mechanistic Convergence

Only reflexes showing a reliable stage 2 signal progress to independently selected mechanistic measurements. Grasp-related changes can be paired with force-matched fNIRS or TMS. Tonic or Moro-like changes can be paired with vestibular, postural, or electromyographic tests. The relevant direction, region, pathway, and weakening criterion are prespecified as outlined in Table 1. Broad exploratory biomarker panels are secondary.

### 6.4. Stage 4—Incremental and Prognostic Value

After replication of a convergent signal, models should test whether adding reflex information improves calibration, discrimination, or clinically meaningful prediction beyond symptoms, SCAT6, VOMS, balance, exercise tolerance, and established covariates [1,2,33]. Internal validation, penalization where appropriate, and independent external validation are required before any prognostic interpretation.

### 6.5. Stage 5—Intervention Testing

**Figure 1:**
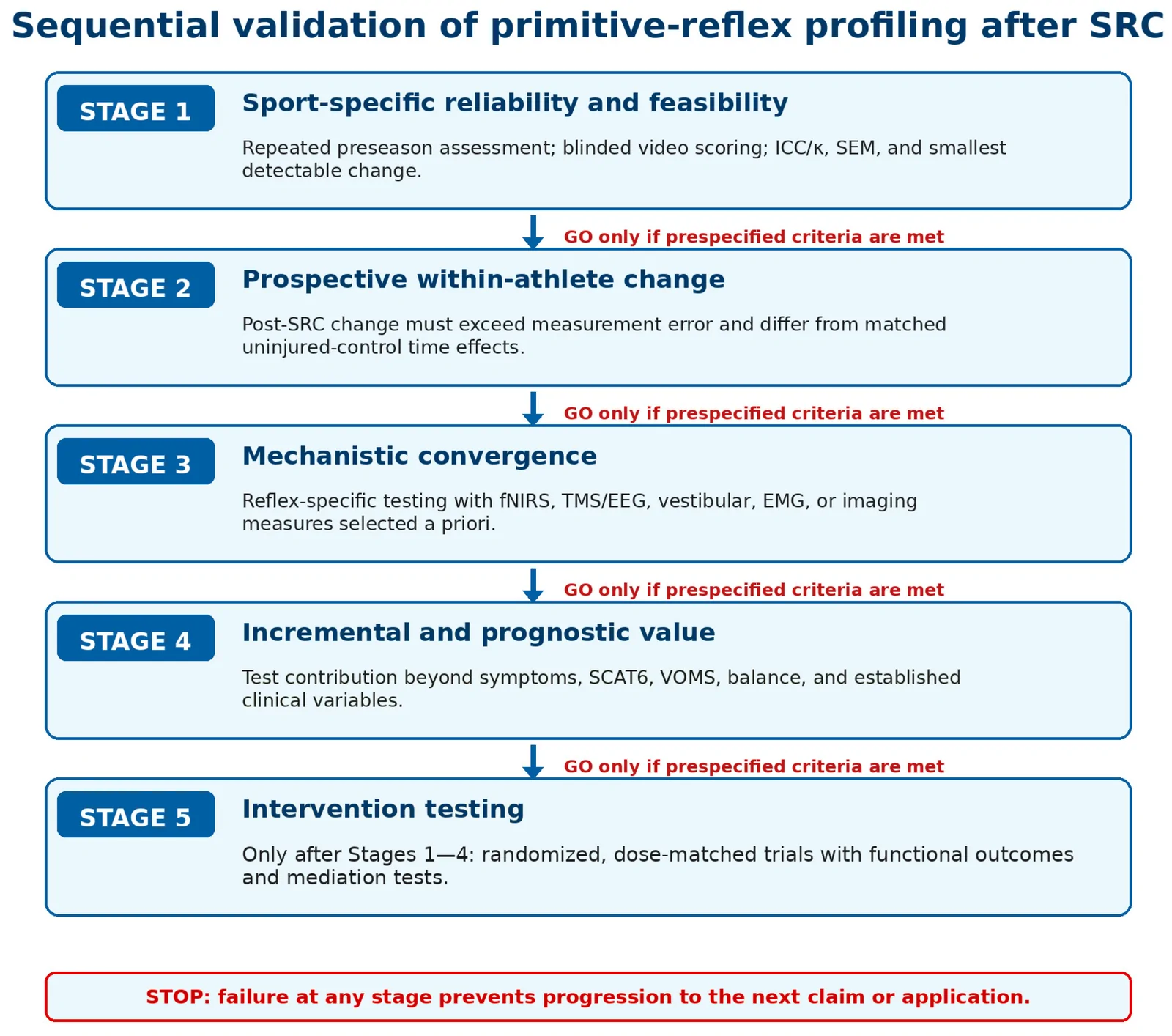
Stage-gated validation program. Progression requires prespecified criteria at each stage; failure stops escalation to the next level of claim. Stage 1 criteria are defined before outcome inspection, clinical safety governs stage 2, and unperformed tests are recorded as missing.

Only after prespecified criteria have been met at stages 1–4 should a reflex-informed intervention be tested in a randomized, assessor-blinded, dose-matched design. Functional recovery—not reflex suppression alone—is the primary clinical target, and mediation analysis should test whether reflex change plausibly links the intervention to function. The complete stage-gated validation sequence and its prespecified stopping logic are summarized in Figure 1.

## 7. Prespecified Predictions and Decision Rules

Interpretation should be governed by decision rules established before outcome inspection. Examples of these stage-specific decision rules are summarized in Table 3 and should be operationalized with numerical thresholds, confidence intervals, multiplicity control, and a registered analysis plan in each study.

## 8. Neurorehabilitation Hypotheses and Boundaries

Exercise can alter cortical metabolites and neurotrophic signaling [34,35], and early subthreshold aerobic exercise can improve clinical recovery after SRC [36]. Those observations support active rehabilitation broadly. They do not demonstrate that reflex-directed exercises normalize a primitive reflex or mediate recovery. Exploratory studies in older adults report associations with or changes in primitive-reflex measures [37,38], but differences in age, study design, and clinical context preclude direct extrapolation to athletes with SRC.

If earlier validation stages succeed, two mechanistically distinct intervention hypotheses can be compared. A bottom-up program would use standardized repeated developmental movement patterns to alter afferent feedback and spinal, brain-stem, vestibular, or cerebellar gain. Its predicted mediators are reflex-specific stimulus–response gain, vestibular–postural integration, and stimulus-locked electromyography. A top-down program would train intentional movements that are incompatible with the elicited synergy to engage cortical selection, intersegmental dissociation, and voluntary inhibition. Its predicted mediators are improved task-specific suppression, premotor/SMA lateralization, and transfer to voluntary or dual-task motor control.

These hypotheses require randomized comparison with attention- and dose-matched active controls, blinded outcome assessment, adverse-event reporting, treatment-fidelity checks, and preregistered functional outcomes. Because the existing multimodal clinical report cannot isolate the reflex-related component [12] and the broader review literature does not establish efficacy [13], no reflex-specific rehabilitation recommendation is made here. The therapeutic target remains clinically meaningful improvements in symptoms, participation, and function. Normalization of a reflex score is at most a candidate mediator or secondary outcome.

## 9. Limitations and Falsification Criteria

The central limitation is the lack of controlled prospective evidence that primitive-reflex expression changes after SRC. The present framework cannot establish that such change exists, that a behavioral response reflects a particular circuit, or that modifying the response improves recovery. Evidence from focal lesions, more severe traumatic brain injury, developmental neuroscience, aging, TMS, fNIRS, or animal models is used only to derive testable predictions. None is a substitute for an SRC cohort with pre-injury data.

Further limitations include ordinal scoring, possible floor effects in healthy athletes, dependence on examiner technique, heterogeneity among the 18 reflexes, multiple testing, unmeasured pain or arousal, and uncertain generalizability across age, sex, sport, concussion history, and time since injury. A summed PRB score may conceal opposing or unrelated reflex changes, and fNIRS or TMS findings may be compensatory, epiphenomenal, or driven by systemic and task-related factors.

The hypothesis should be abandoned or materially revised if athlete-specific reliability is inadequate, post-SRC change does not exceed measurement error or matched-control drift, change lacks recovery coherence, reflex-specific physiological predictions fail repeatedly, incremental value is absent in validated models, or independent teams cannot replicate the findings. Publication of null findings and protocol deviations is essential to prevent a flexible, non-specific examination from becoming self-confirming.

## 10. Conclusions

Primitive-reflex profiling after SRC is a hypothesis-generating research strategy, not a validated biomarker or clinical decision tool. Its scientific value depends on a strict sequence: establish athlete-specific measurement properties, demonstrate prospective within-athlete change beyond error, test reflex-specific neural mechanisms with independent modalities, quantify incremental value, replicate the findings independently, and only then examine intervention sensitivity. This stage-gated design converts an intriguing, but currently weakly evidenced idea into a program in which negative results are informative. Until those tests succeed, guideline-concordant multimodal assessment and rehabilitation remain the standard of care.

## Figures and Tables

**Table 1:** Mechanism-specific predictions and findings that would weaken each account.

Candidate Account	Expected Pattern If Supported	Finding That Weakens the Account
Fronto-subcortical stopping network	Within-athlete grasp change plus the prespecified fronto-subcortical deviation; primary fNIRS prediction: reduced contralateral task-evoked HbO during force-matched grasping. TMS and connectivity are independent tests; co-recovery adds support.	Reliable grasp change without independent fronto-subcortical deviation or temporal coupling. Precise null or opposite-direction HbO findings weaken the primary fNIRS prediction; additional recruitment alone does not confirm it.
Sensorimotor/afferent gating	Reflex-specific change in threshold, gain, stimulus-locked EMG, cutaneous/H-reflex modulation, or SAI/LAI.	Normal, stable afferent-gating and reflex-gain measures despite behavioral change.
Vestibular–reticulospinal regulation	Head-position or startle-related reflex change coupled with VOR/VOMS, head-turn EMG, sensory-integration balance, or startle/postural abnormalities.	Stable vestibular–reticulospinal measures while only cortical variables change.
Non-specific/measurement account	Examiner, pain, fatigue, arousal or time effects explain scores; similar changes in controls; poor retest or video agreement.	Stable blinded measurement, injury-specific within-athlete change, reflex specificity and independent physiological convergence.

**Table 2:** Operational overview of the 18-reflex examination battery described in the source protocol [30,31].

	Reflex Label	Position	Standardized Eliciting Maneuver (Abridged)	Laterality of Recording
1	Asymmetric tonic neck	Standing	Passive head rotation to one side	Bilateral
2	Hip–lumbar Galant	Quadruped	Draw a blunt object along one side of the spine	Bilateral
3	Symmetric tonic neck	Quadruped	Passive head flexion and extension	Bilateral
4	Tonic labyrinthine—forward	Supine	Change head position relative to gravity	Bilateral
5	Tonic labyrinthine—backward	Prone	Change head position relative to gravity	Bilateral
6	Moro	Standing	Sudden, controlled head drop	Bilateral
7	Parachute	Standing	Controlled forward bending with swinging movement	Non-lateralized
8	Palmar grasp	Sitting	Pressure to the palm	Bilateral
9	Plantar grasp	Sitting	Pressure to the plantar surface	Bilateral
10	Babinski	Sitting	Stroke the lateral plantar surface	Bilateral
11	Babkin–palmomental *	Sitting	Firm palmar pressure with active finger movement	Bilateral
12	Rooting	Sitting	Perioral tactile stimulation	Bilateral
13	Plantar withdrawal	Sitting	Stroke the outer edge of the sole	Bilateral
14	Diving	Supine	Apply a cold, wet cloth between the nose and eyebrows	Non-lateralized
15	Fear paralysis	Supine	Sudden unexpected auditory stimulus	Non-lateralized
16	Snouting	Sitting	Lightly tap or stroke the center of the upper lip	Non-lateralized
17	Glabellar	Sitting	Tap the glabella	Non-lateralized
18	Perez	Quadruped	Draw a blunt object bilaterally along the spine	Non-lateralized

* The compound label is retained exactly as used in the source protocol. All labels in this table denote protocol-defined research examinations. They should not be assumed to be interchangeable with every similarly named neurological sign or to share a common mechanism. Scoring must follow the operational motor-response criteria in the complete protocol [31]. This table summarizes source-protocol maneuvers and does not imply that all examinations should be administered during acute SRC. Clinical screening and protocol-specific contraindications govern administration. Unsafe maneuvers must be deferred and recorded as missing.

**Table 3:** Examples of stage-specific findings that support, weaken, or stop the proposed research program.

Question	Finding That Supports Progression	Finding That Weakens or Stops Progression
Reliability and feasibility	Reflex- and side-specific reliability and feasibility meet thresholds defined before outcome inspection, with adequate precision; SEM/SDC are estimable and no unacceptable safety signal is present.	Poor agreement, extreme floor effects, unstable scoring, unacceptable missingness or safety concerns → revise or stop.
Within-athlete injury change	Safely obtained, comparable post-SRC measurements show change exceeding SDC and differing from time-matched control change.	Observed change within measurement error or equally present in controls does not establish a state-marker signal. Unsafe or unperformed tests are missing and cannot establish or refute such a signal.
Recovery trajectory	Reflex change moves toward baseline with recovery and its confidence interval excludes expected retest drift.	No temporal coherence, inconsistent direction, or worsening unrelated to recovery → weaken state interpretation.
Mechanistic convergence	The prespecified reflex-specific physiological change co-occurs and co-recovers with behavioral change. For grasp-related fNIRS, contralateral HbO attenuation is primary; lateralization is secondary.	Adequately precise null or reproducible opposite-direction findings weaken the specified prediction. Increased HbO is an alternative requiring separate evaluation; imprecise estimates remain inconclusive.
Specificity and laterality	Pattern matches the eliciting condition, reflex, side and candidate pathway.	Only the total PRB changes, or unrelated reflexes/sides change diffusely → favor non-specific explanation.
Incremental value	Validated model improves performance beyond established clinical variables with useful calibration and external replication.	No out-of-sample improvement or poor calibration → no prognostic claim.
Intervention sensitivity	Randomized functional benefit accompanies predicted reflex and mechanistic change; mediation is compatible with the model.	Reflex suppression without functional benefit, no group effect, or benefit without predicted mediation → no mechanism-based treatment claim.

## Data Availability

No new data were created or analyzed for this Perspective. The complete standardized primitive-reflex examination protocol—including detailed administration procedures, operational response criteria, safety adaptations, demonstration materials, and examiner guidance—is openly available through Mendeley Data [31].

## Abbreviations

BOLD, blood oxygen level–dependent; EEG, electroencephalography; fMRI, functional magnetic resonance imaging; fNIRS, functional near-infrared spectroscopy; GABA, γ-aminobutyric acid; HbO, oxygenated hemoglobin; HbR, deoxygenated hemoglobin; ICC, intraclass correlation coefficient; LAI, long-latency afferent inhibition; PRB, primitive-reflex burden; SAI, short-latency afferent inhibition; SCAT6, Sport Concussion Assessment Tool 6; SDC, smallest detectable change; SEM, standard error of measurement; SMA, supplementary motor area; SRC, sport-related concussion; TMS, transcranial magnetic stimulation; VOMS, vestibular–ocular motor screening; VOR, vestibular–ocular reflex.
